# Genome and Transcriptome Sequences of *Pseudomonas syringae* pv. syringae B301D-R

**DOI:** 10.1128/genomeA.00306-14

**Published:** 2014-04-10

**Authors:** Alexey Dudnik, Robert Dudler

**Affiliations:** Institute of Plant Biology, University of Zurich, Zurich, Switzerland

## Abstract

Strains of the plant pathogen *Pseudomonas syringae* are commonly found in the phylosphere and are able to infect a number of agriculturally important crops. Here, we report a high-quality draft genome sequence of *Pseudomonas syringae* pv. syringae B301D-R, isolated from pears, which is a model strain for phytotoxin research in *P. syringae*.

## GENOME ANNOUNCEMENT

*Pseudomonas syringae* is a highly diverse plant pathogen with significant economic and environmental impacts. It is also an important model for plant-pathogen interaction research (1). In order to suppress host defense responses and to promote disease symptom development, the pathogen utilizes type III-translocated effectors (T3Es), as well as a variety of other secreted substances, such as phytotoxins, exopolymeric compounds, phytohormones, etc. (2–5). *P. syringae* pv. syringae B301D-R is a spontaneous rifampin-resistant mutant of a strain isolated from diseased pears (*Pyrus communis*) in England (6). *P. syringae* B301D was used in a number of studies, some dealing with the production and importance of the phytotoxins syringomycin, syringopeptin, and syringolin (7–16).

An 800-bp Nextera XT library was generated and sequenced at Microsynth AG using the Illumina MiSeq platform. A total of 2,404,408 quality filtered reads with a total of 594,414,359 bases were obtained, resulting in 98.5-fold average sequencing coverage. The obtained reads were further *de novo* assembled using CLC Workbench 6.0.1 into 81 contigs encompassing 6.04 Mbp in total. Automatic open reading frame (ORF) prediction and functional annotation have been performed with Prokka 1.8 (17) using non-redundant protein sequence (nr) and custom databases.

The assembly size for *P. syringae* B301D-R is 6,036,561 bp, with an average G+C content of 59.2%. It contains 5,185 protein-coding sequences, 54 tRNA genes for all 20 amino acids, and 29 noncoding RNA genes. The genome contains a complete *hrc*/*hrp* family type III secretion system and genes for twelve known T3Es: HopM1, HopI1, HopAE1, HopAA1, HopAG1, AvrE1, HopAH1, HopAL1, HopH1, HopA2, HopAI1, and HopBC1. Moreover, it contains two complete type VI secretion system gene clusters and twelve putative type VI effector-coding genes: seven of the VgrG type and five of the Hcp1 type. The genome sequence completely covers the syringolin biosynthesis gene cluster (PssB301D_04806 to PssB301D_04810), as well as most of the syringopeptin and syringomycin biosynthetic genes, with the exception of sequences encoding parts of nonribosomal peptide synthetases, which are difficult to assemble using short reads. A mangotoxin biosynthesis operon, commonly found among phylogroup II strains (18), was not detected. B301D-R also contains genes required for production of exopolysaccharides alginate, Psl, and levan.

In addition, we have generated transcriptome data for the wild type B301D-R, as well as for its *salA*-deficient derivative DSL7 (19) from cells grown on solid SRM_AF_ medium (10) for 72 h at 18°C. Total RNA isolates from three independent experiments were combined together and sequenced using the Illumina MiSeq platform at Microsynth AG. The *salA* gene encodes a transcriptional regulator that, among other functions, controls phytotoxin production in *P. syringae* (14, 19). So far, only limited microarray data are available for this mutant (8), and therefore, whole-transcriptome data allow the uncovering of the complete regulon of SalA. Sequencing reads were deposited at the NCBI Sequence Read Archive (SRA) under accession no. SRP035451.

### Nucleotide sequence accession numbers.

This whole-genome shotgun project has been deposited at DDBJ/EMBL/GenBank under the accession no. JALJ00000000. The version described in this paper is the first version, JALJ01000000. The assigned NCBI taxonomy identification number is 1365665.
